# Visualizing an Ethics Framework: A Method to Create Interactive Knowledge Visualizations From Health Policy Documents

**DOI:** 10.2196/16249

**Published:** 2020-01-14

**Authors:** Joanna Sleigh, Manuel Schneider, Julia Amann, Effy Vayena

**Affiliations:** 1 Health Ethics and Policy Lab Department of Health Sciences and Technology ETH Zurich Zurich Switzerland

**Keywords:** ethics framework, health data, health policy, knowledge visualization, systems map

## Abstract

**Background:**

Data have become an essential factor in driving health research and are key to the development of personalized and precision medicine. Primary and secondary use of personal data holds significant potential for research; however, it also introduces a new set of challenges around consent processes, privacy, and data sharing. Research institutions have issued ethical guidelines to address challenges and ensure responsible data processing and data sharing. However, ethical guidelines directed at researchers and medical professionals are often complex; require readers who are familiar with specific terminology; and can be hard to understand for people without sufficient background knowledge in legislation, research, and data processing practices.

**Objective:**

This study aimed to visually represent an ethics framework to make its content more accessible to its stakeholders. More generally, we wanted to explore the potential of visualizing policy documents to combat and prevent research misconduct by improving the capacity of actors in health research to handle data responsibly.

**Methods:**

We used a mixed methods approach based on knowledge visualization with 3 sequential steps: qualitative content analysis (open and axial coding, among others); visualizing the knowledge structure, which resulted from the previous step; and adding interactive functionality to access information using rapid prototyping.

**Results:**

Through our iterative methodology, we developed a tool that allows users to explore an ethics framework for data sharing through an interactive visualization. Our results represent an approach that can make policy documents easier to understand and, therefore, more applicable in practice.

**Conclusions:**

Meaningful communication and understanding each other remain a challenge in various areas of health care and medicine. We contribute to advancing communication practices through the introduction of knowledge visualization to bioethics to offer a novel way to tackle this relevant issue.

## Introduction

We live in an era where data are omnipresent and seemingly omnipotent. Data constitute one of the forces, if not *the* driving force, behind personalized and precision medicine. Traditional health data sources such as medical records and clinical trial data are nowadays complemented by an ever-increasing amount of behavioral and lifestyle data, which we create by interacting with everyday technologies such as our smartphones. Although data collection remains important, data sharing is fundamental to modern scientific practice and is of great value to the health sciences. First, this is because data are crucial to the confirmation of research findings and the replication of results [1]. Second, making data available enables scientists to collaborate and build on the work of others [2]. Third, reusing data enables researchers to leverage research investments, particularly public funding [3]. Fourth, data sharing is integral to the advancement of research and innovation [4]. In the health sciences specifically, data sharing has the potential to transform health care and inform clinical research; quality measurement; and, ultimately, public safety [5].

However, although the quantity and types of data available for research are rapidly expanding, the handling of such data is a complex process that involves and impacts several stakeholder groups such as patients and research institutions. In recognition of precision medicine’s reliance on big data, the Swiss Personalized Health Network (SPHN) produced the Ethical Framework for the Responsible Usage of Personal Data in Health Research [6]. The framework guides SPHN’s actors (such as researchers) as they endeavor to handle data ethically, to inform research participants about these ethical practices, and to tackle concerns regarding privacy and misuse of data. However, the SPHN ethical framework is innately complex. Like many health policy documents, the SPHN framework describes a multilayered, nonlinear process that involves several stakeholders. The problem with this is that the document needs to refer to other elements of the framework to cover 1 aspect fully [7]. For example, the process of consent involves the research participant, the researcher, and the institution. In addition, it is a process interwoven at different stages of a research project, from the very beginning to long after the project has finished.

Ethical and policy guidance is only as effective as its application. To combat and prevent research misconduct and to foster data sharing practices, stakeholders involved need to understand available guidance and apply it in practice. Some researchers have questioned the efficacy of policy documents as communication tools [8]. Research shows that senior decision makers often do not read long policy documents [9]. However, senior decision makers are not the only critical audience for health policy. For example, the SPHN ethical framework is relevant to diverse members of the SPHN network, from researchers to medical practitioners and individuals who participate in studies. Policy documents are no match to the challenge of communicating complex information to tremendously diverse audiences [10], and well-intentioned stakeholders can find it onerous to act on the information in the document. Whenever this happens, policy documents defy their purpose.

So how can we ensure that policy documents actually fulfill their purpose? Extensive research exists on how to communicate complex information to stakeholders. One promising approach is knowledge visualization. Knowledge visualization “examines the use of complementary visual representations to improve the transfer and creation of knowledge between at least two persons” [11]. Evidence indicates that the active integration of visual representations improves learning significantly [12,13]. Theories that support this include Paivio’s dual coding theory [14], which asserts that we process verbal information and pictorial information in different cognitive systems, as well as Chandler and Sweller’s cognitive load theory [15], which argues that multiple sources of information facilitate learning by reducing working memory. These insights and opportunities are currently not used to inform knowledge dissemination in health policy making [10,16,17]. To address this gap, we adopted a knowledge visualization methodology and applied a mixed methods approach to translating an existing ethical framework into an interactive knowledge visualization tool. In this study, we present our methodological approach and describe the process that led us to this method. To do this, we take the SPHN ethical framework for the Responsible Usage of Personal Data in Health Research as a case study.

## Methods

### Overview

**Figure 1:**
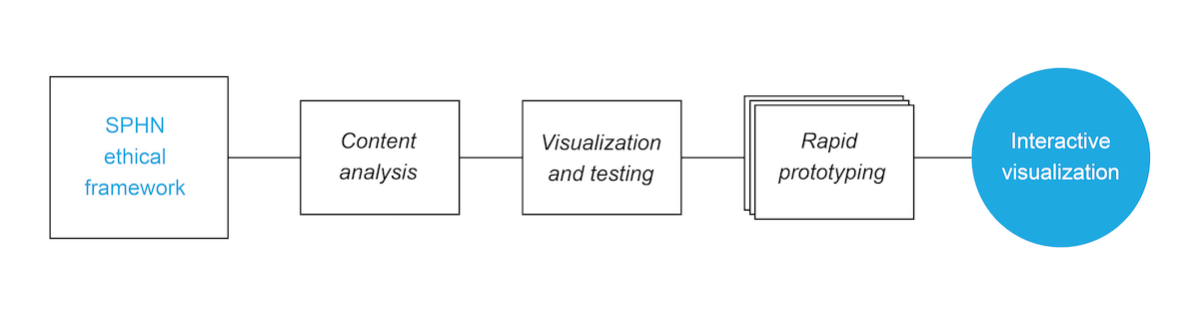
This figure gives an overview of the method and outlines the 3 primary steps: content analysis, visualization, and rapid prototyping. The rectangle on the left represents the Swiss Personalized Health Network’s ethical framework in its original form as a document, and the circle on the right represents the interactive visualization of the framework’s content. SPHN: Swiss Personalized Health Network.

Our approach to transforming a complex framework into an interactive visualization involved 3 steps, as shown in Figure 1. To begin with, we conducted a quantitative content analysis to distill and structure the knowledge inside the document. We used a combination of inductive and deductive methods to create a conceptual representation of the content sequentially. In a second step, we transferred the conceptual data into 4 different visual forms and, then, tested these visualizations through expert review to select the most promising candidate. Although the visualizations did already make the content of the framework more accessible, the interwoven structure of the knowledge was still not entirely represented. In a final step, we, therefore, iteratively developed an interactive version of the visualization through rapid prototyping. We look now at each of these steps in more detail.

### Content Analysis

Our first step was to identify the knowledge and knowledge structure manifested in the framework. To do this, we analyzed the framework using a qualitative content analysis approach that combined inductive and deductive category development [18-20]. Moreover, 1 author (JS) with training and previous experience in inductive and deductive coding performed the analysis. We ensured intracoder reliability through 3 rounds of coding, with the first 2 within a month and the third round 3 months later. We resolved any discrepancies in discussions within the research team.

We conducted an inductive analysis to identify (1) the key elements in the ethical framework, (2) stakeholders, (3) the knowledge types present in the SPHN framework, and (4) the connections between the elements and stakeholders. We started with open coding, with the restriction that codes had to be mutually exclusive to ensure that the resulting knowledge structure remains unambiguous [21].

We then grouped codes that concerned the same subject matter and merged them into categories. To give an example, we merged the codes *withdraw* and *revoking consent* into *withdrawal process* just as we merged *communication* and *information* into *participant information*. These categories form the lowest layer of abstraction in the knowledge structure of the framework, that is, the subthemes. This task was conducted iteratively, where we revised, refined, and checked the subthemes to ensure that they remained mutually exclusive.

In the next step, we formed overarching themes out of the subthemes, adding another layer of abstraction. As an example, the 2 subthemes *further use* and *withdrawal process* both belong to the theme *consent process*. These groups can overlap as a subtheme can belong to multiple groups. For example, *further use* is part of both the *consent process* and the *data and samples* themes.

We then determined the primary stakeholders from the frequency of their occurrences in the text and used axial coding to identify and map out the relationships between the stakeholders, themes, and subthemes [22]. Although some of the coded elements explicitly indicated relationships among each other and with stakeholders, we had to infer others from context.

Finally, we used deductive analysis to assign the stakeholders, themes, subthemes, and their relationships with 1 or more of the 4 normative ethical principles of the SPHN framework: respect for persons, data fairness, privacy, and accountability. The resulting 4 groups are not mutually exclusive as a stakeholder, theme, and subtheme can be affected by several ethical principles. This fact contributed to the complexity of the original SPHN framework document.

A total of 3 content experts from the fields of bioethics (EV and AB) and public health (FG) reviewed the coding and the knowledge structure. We selected the experts through purposive sampling. Moreover, 2 of the 3 experts (EV and AB) contributed to the development of the original SPHN framework and made sure that the result reflects the entirety of the framework’s content.

Multimedia Appendices 1-4 present the results of the inductive and deductive analysis.

### Visualization Methods

To visualize the previously derived knowledge structure in a simple yet comprehensive way, we tested different graphics and visualization methods: alluvial diagrams, graphics such as symbols, concept maps, and systems maps. The 4 methods were chosen based on Burkhard’s model of visualization types for knowledge visualization [23]. The following paragraphs describe the visualization methods. See Multimedia Appendix 5 for the outputs.

#### Alluvial Diagram

Alluvial diagram is a type of flow diagram, or branch-based diagram, that represents weighted correlations between categorical dimensions, visually linking the number of elements to shared categories [24]. As abstract and schematic representations, alluvial diagrams are used to explore structural relationships among parts and are, therefore, used to explain concepts and reduce complexity. For this reason, we first employed an alluvial diagram to visually explore the various relationships among themes, actors, and ethical principles.

#### Signs, Symbols, and Sketches

Sketches, drawings, symbols, and icons are nonverbal representational forms used for knowledge transfer and communication. The making of meaning from visual representations is a very different undertaking than that of language [25]. Sketches are, thus, a useful and powerful visualization tool that enables quick communication and that stimulates creativity by leaving room for interpretation [26]. For these reasons, we transmediated the key themes and subthemes into a series of icons and visual metaphors to retain user attention, enhance understanding, and improve recall.

#### Concept Map

Concept mapping, also referred to as structured conceptualization, is an established method for the organization and representation of knowledge. The method produces a map that consists of nodes and lines—the nodes indicating concepts and the connecting lines denoting relationships between them [27]. What differentiates concept mapping from methods such as mind mapping, cluster mapping, and flow charts is that concept mapping uses a top-down structure to show the relationships between themes and subthemes with the overall concept. In this study, we chose concept mapping to visualize the ethical framework’s 4 core ethical principles and the actors, issues, and concepts that relate to them.

#### Systems Map

Systems mapping is the process of visually representing and describing an entire system, including the elements and actors involved as well as their relationships, links, and interconnections. This method makes clear how things such as information or materials flow through a system. Other types of systems maps are causal loop diagrams, actor-network maps, and value chain maps. Reasons for using this method are as follows: (1) systems maps make sense of complexity, (2) they engage stakeholders by highlighting their position in the system, and (3) they enable both issues and opportunities to be easily identified [28]. We chose this method to visualize the entirety of the ethical framework’s knowledge system to make clear the patterns of process and underlying relationships between values and beliefs (mental models) of the actors (people and organizations) involved, responsible and impacted.

### Testing Through Expert Review

We invited 2 experts (EV and AB) to assess which visualization method was most appropriate for the SPHN framework content. Both experts were selected because they had contributed to the revision of the original SPHN framework in 2018 and the underlying analysis of existing policies [29]. We conducted the expert reviews as informal interviews, wherein we presented the 2 reviewers each of the tested visualizations to determine their respective strengths and weaknesses. To do so, we structured interviews based on Burkhard’s knowledge visualization framework [23]:

1. Attention: Is the visualization attractive and engaging?
2. Context: Does the visualization convey why the knowledge is needed and is of value?
3. Overview: Does the visualization give an overview of the complexity of the framework?
4. Options to act: Does the visualization provide options to act, to use, and to apply the knowledge.
5. Details: Is the amount of detail appropriate?

The experts’ review showed that although many of the visualization methods were engaging, they oversimplified the content, making important details inaccessible. For example, the concept maps were determined to be effective at showing the actors and elements inherent to each ethical principle, but neither did they explain how the principles related to each other nor did they provide definitions. Only the system map was successful in providing both an overview and adequate detail of the ethical framework. However, according to the experts, the systems map failed to make the underlying ethical principles visible. Despite some shortcomings, we identified the systems map to be the most appropriate method.

### Rapid Prototyping

To address the shortcomings of the systems map and make the SPHN ethical framework content accessible in its entirety, we developed an interactive visualization using rapid prototyping [30]. We wanted to enable users to explore across registers, from the big concepts to the specific details in the ethical framework’s range of knowledge types (declarative, experiential, individual, orientational, and procedural). This goal was informed by Ausubel’s assimilation theory [31]. We, hence, devised a Web-based system map that proceeded from the more general, more inclusive concepts to the more specific information.

To assess the design and usability of the prototype, we conducted 3 expert reviews (by DG, IS, and ML) at different stages of the prototyping process with a different reviewer for each stage. We used convenience sampling to find experts for user experience (UX) research, design, and storytelling. For this purpose, we presented reviewers with the prototype and asked them to provide feedback. A UX researcher, with a background in media studies and economics, participated in the first expert review. A UX designer with a Masters in Design and over 15 years of experience in product development undertook the second review. This expert was familiar with the topic of data processing, yet had no experience in the health sector. A UX copywriter completed the final review. This reviewer has a background in nanotechnology design and is also familiar with data processing and data sharing practices. These experts judged the prototypes according to usability requirements and according to the previously introduced questions by Burkhard [23]. We systematically recorded and compared the comments and suggestions for improvement received from each of the 3 experts to inform the next prototype iteration.

To prototype an interactive systems map, we used a data visualization platform called Kumu [32]. We first loaded the identified themes, subthemes, and stakeholders into Kumu and designated the nodes by labels and size. Large nodes represented stakeholders, medium-sized nodes indicated themes, and we gave small nodes to subthemes. We then clustered and linked these nodes according to theme and subtheme hierarchies and the relationships identified from axial coding. Afterward, we pollinated the nodes with metadata and descriptions derived directly from the SPHN ethical framework. We simplified the wording at times or added definitions from the SPHN ethical framework glossary document. Concurrently, we embedded the visual icons developed during the initial testing phase to support content recall for users. For the primary 11 themes (consent, upholding human rights, authorization procedures, governance structures, data + sample processing, accountability processes, security control processes, scientific research, data and samples, transparency, and sharing process), we animated these line icons into gifs that loop.

Expert reviewers noted some limitations with Kumu; thus, to overcome these, we developed a website with a customized interactive visualization of the nodes with additional functionality and information. In addition, we implemented custom views that present content based on the different stakeholders’ perspectives on the systems map. Each perspective was composed of 11 views that highlight the themes and explain their importance according to the respective stakeholder’s responsibilities and interests. Furthermore, we integrated the 4 ethical principles by allowing the user to highlight the affiliation of the nodes to the activated principle.

## Results

In this section, we present the final interactive visualization prototype of the SPHN ethical framework and its features. In total, we identified 4 primary stakeholders, 3 general themes, 8 research process themes, 2 effect themes, and 30 subthemes. The inductive analysis further revealed that the SPHN ethical framework comprises multiple knowledge types. These included the following: declarative knowledge (know-about), experiential knowledge (know-why, eg, causes), individual knowledge (know-who), orientational knowledge (know-where), and procedural knowledge (know-how) [33]. Multimedia Appendices 1-5 present the results of intermediate steps in more detail.

The focal point of the prototype is the interactive systems map visualizing the SPHN ethical framework. It shows the stakeholders, themes, subthemes, and their relations to each other. The map is made up of edges and nodes. The large-sized nodes correspond to the stakeholders, whereas the medium-sized nodes represent the themes and the small-sized nodes indicate the subthemes. In addition to the map itself, there are complementary functionalities, which we describe along with the other interaction techniques in the following paragraphs.

**Figure 2:**
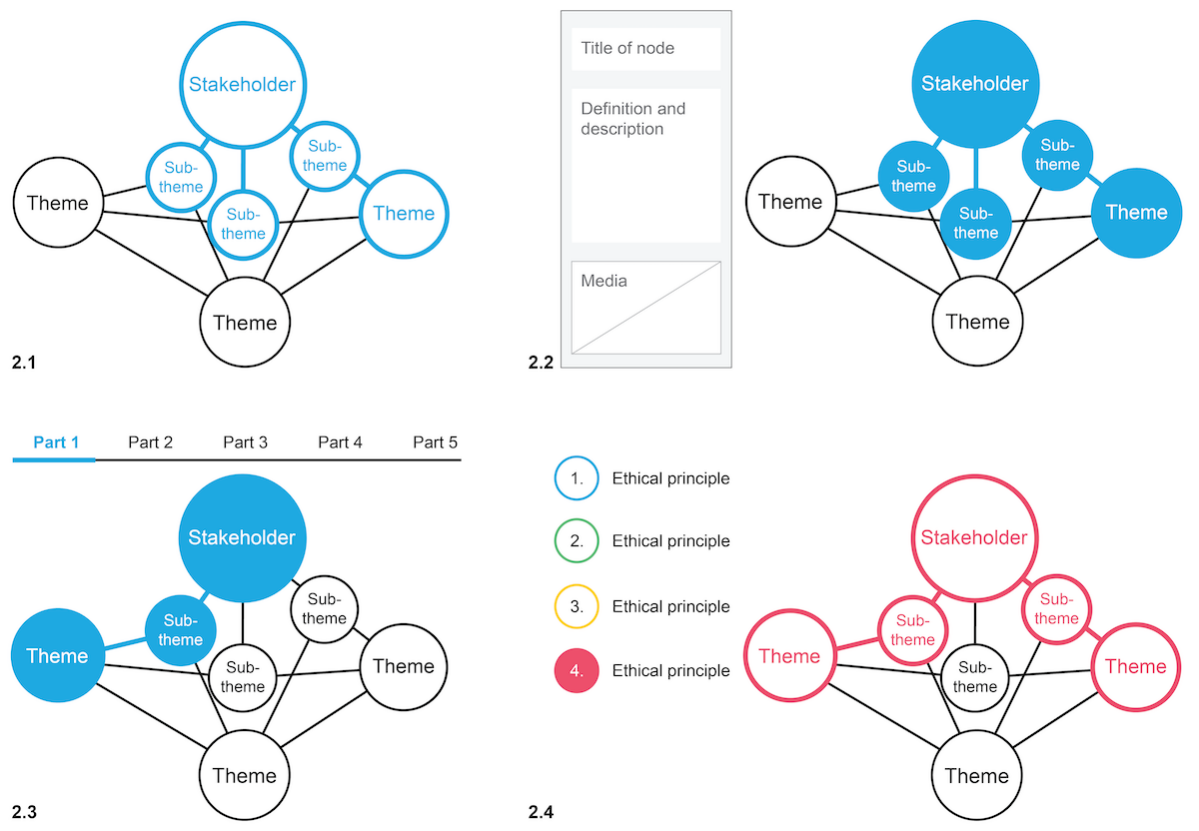
In image 2.1, we see the effect of hovering over a node that highlights connections. Image 2.2 shows the function of clicking to access information and metadata. Image 2.3 depicts the buttons that represent the stakeholder perspectives and the node highlighting in the map below as response when one of them is activated. Image 2.4 demonstrates the function of highlighting ethical principles using the color legend simultaneously to the other functions where each color corresponds to an ethical principle.

First, users can hover to highlight a node and its connections (see Figure 2). This functionality is integral for highlighting the interconnectedness of the themes and stakeholder.

**Figure 3:**
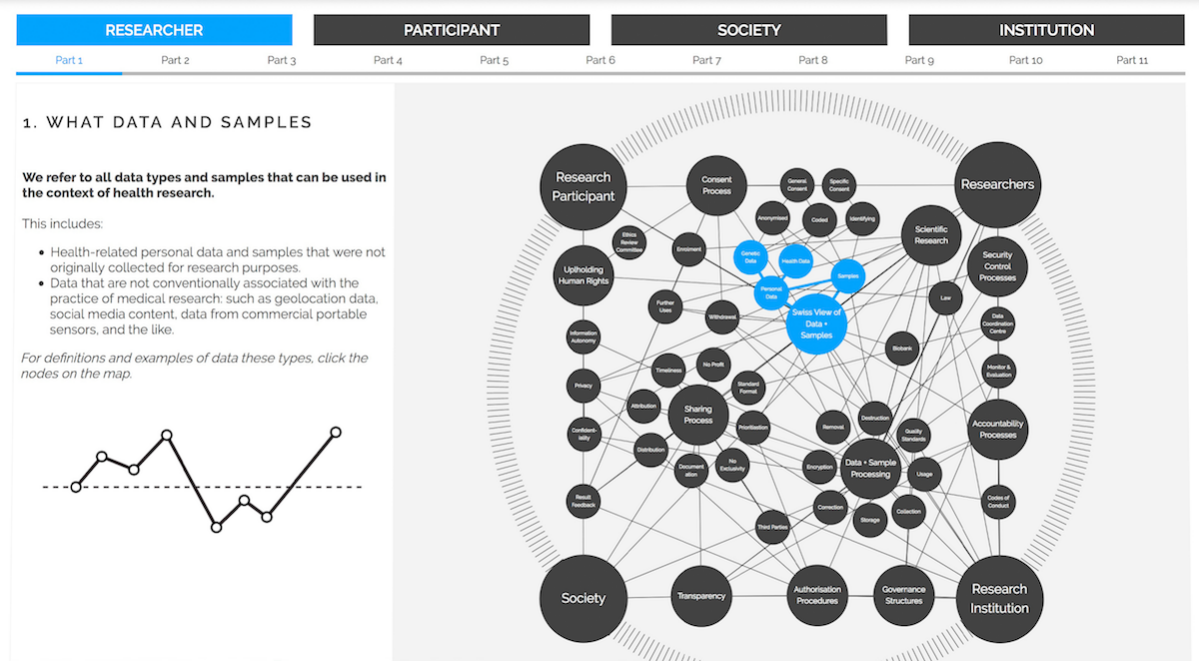
Screenshot of the interactive systems map.

Second, the select function is used to activate the display of a node’s information and metadata. In other words, by clicking on a node, users can access the information based on the SPHN ethical framework (see Figure 2). Each node is made up of a title, a definition or description of what it refers to, information about why it matters, and references to the connected nodes. In addition, to make the information easier to understand, each node includes embedded explanatory media such as animated gifs, videos, and graphics. Figure 3 exhibits a screenshot of the final interactive visualization [34] (additional screenshots are provided in Multimedia Appendix 6).

Third, to enable navigation of the content according to the stakeholder categorizations, stakeholder perspectives were defined and can be activated through buttons above the map. Each stakeholder’s perspective is divided into 11 subperspectives, titled parts, reflecting the 11 themes of the respective perspective. By clicking on a perspective and subperspective button, for example, *Researcher* and *Part 1*, the nodes corresponding to the subperspective are highlighted in the map, and additional information for the selection is displayed (see Figure 2). These perspectives enable a linear navigation approach to the clusters and connections of the systems map. We further adjusted the language style and level of detail according to the stakeholder’s respective responsibilities and interests. For example, the society viewpoint for *data governance* is less detailed than the institution viewpoint for the same topic.

Fourth, to incorporate the SPHN ethical framework’s 4 ethical principles, we used a color legend. As such, we assigned each ethical principle a color and tagged the nodes accordingly. By clicking an ethical principle in the legend, users can then highlight the relevant nodes (see Figure 2). In this way, users experience the interwoven nature of ethical principles.

## Discussion

### Principal Findings

In this study, we developed an interactive visualization to navigate the content of the SPHN ethical framework. Our mixed methods approach consisted of qualitative analysis, visualization techniques, and rapid prototyping. In contrast to the ethical framework’s original form as a text document, our interactive visualization offers users an overview of the ethical framework content and provides access to detailed descriptions and definitions enriched with multimedia content. Another unique function of our interactive visualization is that it allows users to examine the relationships between elements and themes.

Our method finds application beyond the specific type of policy document we used in our case study. Specifically, the individual parts of the method can be applied independent of context to transform a defined text scope. Only the choice of visualization type based on the expert review was specific to bioethics. This dependency can be resolved by calling in experts from other respective fields. The method presented in this study, therefore, holds great potential for a variety of text contents beyond policy documents and bioethics.

### Limitations

Future research is needed to unlock the potential of this visualization approach. To begin with, this study does not assess the interactive visualization’s educational powers compared with the original policy document. Further research is, thus, required to measure the effectiveness of the knowledge transfer. In addition, systematic user testing is needed to resolve functional shortcomings. The combination of these 2 assessments would optimize the tool’s efficacy and, therefore, improve the impact of data sharing policies on the stakeholders’ actions.

Another limitation is that the visualization does not assist the stakeholders’ understanding of the dependencies between different elements of the framework. In the words of Tufte, understanding “... is to know what cause provokes what effect, by what means, at what rate” [35]. To resolve this, a functionality that highlights cause and effect could be incorporated and tested. For example, a game function that allows the user to remove nodes from the map and then to see how the scenario evolves enables the stakeholders to explore the impact of different elements of the framework in an engaging way.

### Conclusions

This study sheds light on how to develop an interactive visualization from a policy document and lays the foundation to innovate ethical framework dissemination practices on the Web. In practice, this method to translate ethical frameworks gives bioethicists and health care policy makers a tool to communicate complex information to diverse audiences. More broadly, the results offer guidance to researchers, practitioners, and designers who create dynamic visualization for scientific and scholarly communication.

Meaningful communication and understanding each other remain a challenge in various areas of health care and medicine. We contribute to advancing communication practices through the introduction of knowledge visualization to bioethics to offer a novel way to tackle this relevant issue. Our work bears value for every person involved in modern health research: from policy makers who give guidance, to researchers who process data, to the patients who use their smartphones throughout the day and generate data.

## Appendix

Multimedia Appendix 1 Identified framing themes in responsible processing of personal data and samples in health research.

Multimedia Appendix 2 Identified research process themes in responsible processing of personal data and samples in health research.

Multimedia Appendix 3 Identified effect themes in responsible processing of personal data and samples in health research.

Multimedia Appendix 4 Identified stakeholders in responsible processing of personal data and samples in health research.

Multimedia Appendix 5 Summary of expert reviews of the visualizations.

Multimedia Appendix 6 Screenshots of the interactive visualization.

## Abbreviations

SPHN: Swiss Personalized Health Network
UX: user experience
