# Supplementary material for: Visualizing an Ethics Framework: A Method to Create Interactive Knowledge Visualizations From Health Policy Documents
Source: J Med Internet Res. 2020 Jan 14;22(1):e16249. doi: 10.2196/16249 (PMC6996733; doi:10.2196/16249)
Supplement: Multimedia Appendix 1 [file jmir_v22i1e16249_app1.pdf]

	Framing Theme	Knowledge Type	Explanation
1	Scientific Research	*Declarative (what)*	Scientific research is the systematic study of the physical and natural world and it produces knowledge in the form of results and findings.
2	Data + Samples	*Declarative (what)*	Data & Samples refers to all data types (Genetic, Health related) and human biological materials that can be usefully employed in the context of health research. This includes data and samples that are coded, anonymized or identifiable.
3	Transparency	*Declarative (what)*	Transparency is an underlying value of processing data and samples in health research. Therefore, it is key to the consent, processing, sharing and accountability mechanisms.
