# Supplementary material for: Visualizing an Ethics Framework: A Method to Create Interactive Knowledge Visualizations From Health Policy Documents
Source: J Med Internet Res. 2020 Jan 14;22(1):e16249. doi: 10.2196/16249 (PMC6996733; doi:10.2196/16249)
Supplement: Multimedia Appendix 2 [file jmir_v22i1e16249_app2.pdf]

	Research Process Theme	Knowledge Type	Explanation
1	Accountability Processes	*Declarative (what)*<br><br>*Procedural (how)*<br><br>*Experimental (why)*<br><br>*Individual (who)*	Accountability processes, meaning mechanisms and systems of assessment and regulation, must be in place that are fair, lawful and transparent so that research institutions and researchers can be held responsible for the impact of their activities on both research participants and society as a whole.
2	Consent Process	*Declarative (what)*<br><br>*Procedural (how)*<br><br>*Experimental (why)*<br><br>*Individual (who)*	Consent means voluntary and informed authorization of a person to the use of their data and samples for research purposes. The appropriate type of consent must be obtained to ensure research participants rights are protected and that data and samples can be used for research purposes.
3	Data + Sample Processing	*Declarative (what)*<br><br>*Procedural (how)*<br><br>*Experimental (why)*	Data processing means any operation dealing with personal data, irrespective of the means and the procedure employed, and in particular the collection, storage, use, sharing, revision, disclosure, archiving or destruction of such data. Data processing should be fair, lawful and transparent.
4	Data Authorization Procedures	*Declarative (what)*<br><br>*Procedural (how)*<br><br>*Experimental (why)*	Procedures for authorizing access must be standardized, transparent, lean and easily understandable so as to ensure efficient processing of personal data and handling of human biological material.
5	Data Sharing Process	*Declarative (what)*<br><br>*Procedural (how)*<br><br>*Experimental (why)*	Data, samples, and results from research must be appropriately shared and made available for further research use so as to advance the common good of scientific knowledge.

6	Governance Structures	*Declarative (what)*<br><br>*Procedural (how)*<br><br>*Experimental (why)*<br><br>*Orientation (where)*	Governance structures, meaning the framework of rules, relationships, systems and processing by which data and samples are handled, must be in place that are clear, adequate, transparent and auditable.
7	Processes of upholding human rights	*Declarative (what)*<br><br>*Experimental (why)*<br><br>*Orientation (where)*	Human rights mean the right to informational determinism, the right to autonomy, the right to privacy, the right to confidentiality, and the right to be informed. These rights take precedence over the interests of scientific knowledge production and should be respected and protected.
8	Security Control Processes	*Declarative (what)*<br><br>*Procedural (how)*<br><br>*Experimental (why)*<br><br>*Individual (who)*	Security controls, which are safeguards or counter measures, must be in place to protect the integrity of the information system, the privacy of research participants and the confidentiality of their personal information.
