# Supplementary material for: Visualizing an Ethics Framework: A Method to Create Interactive Knowledge Visualizations From Health Policy Documents
Source: J Med Internet Res. 2020 Jan 14;22(1):e16249. doi: 10.2196/16249 (PMC6996733; doi:10.2196/16249)
Supplement: Multimedia Appendix 3 [file jmir_v22i1e16249_app3.pdf]

	Effect Theme	Knowledge Type	Explanation
1	Trust	*Experimental (why)*	Responsible and ethical processing of personal data fosters public trust.
2	Advancement	*Experimental (why)*	Sharing of data and samples is required for scientific progress.
