# Supplementary material for: Visualizing an Ethics Framework: A Method to Create Interactive Knowledge Visualizations From Health Policy Documents
Source: J Med Internet Res. 2020 Jan 14;22(1):e16249. doi: 10.2196/16249 (PMC6996733; doi:10.2196/16249)
Supplement: Multimedia Appendix 4 [file jmir_v22i1e16249_app4.pdf]

	Actors	Knowledge Type	Explanation
1	Regulatory Bodies	*Individual (who)*<br><br>*Procedural (how)*	Regulatory bodies are independent committees that ensure participant rights are respected. They do this by conducting assessments of the research projects and by providing frameworks and standards for the processing of personal data and the handling of human biological material.
2	Research Institutions	*Individual (who)*<br><br>*Declarative (what)*<br><br>*Experimental (why)*	Research institutions are established centers for conducting research. As such they must meet adequate standards of ethical responsibility, promote the rights, interests and well-being of research participants, ensure the efficient production of valuable scientific knowledge, and generate public trust.
3	Researchers	*Individual (who)*<br><br>*Declarative (what)*<br><br>*Procedural (how)*	A researcher is the natural person doing scientific work with the aim to discover new knowledge. A researcher working with personal data or human biological material must be trained on the technical, legal and ethical requirements with regard to data protection.
4	Research Participants	*Individual (who)*<br><br>*Declarative (what)*<br><br>*Procedural (how)*	Individuals contributing data and/or human biological material to research. They have the right to autonomy, privacy, and confidentiality. Their participation should always be both informed and voluntary.
5	Society	*Individual (who)*<br><br>*Declarative (what)*<br><br>*Procedural (how)*	Society, meaning the public in general, should never be negatively impacted by the activities of researchers and research institutions.
