# Supplementary material for: Visualizing an Ethics Framework: A Method to Create Interactive Knowledge Visualizations From Health Policy Documents
Source: J Med Internet Res. 2020 Jan 14;22(1):e16249. doi: 10.2196/16249 (PMC6996733; doi:10.2196/16249)
Supplement: Multimedia Appendix 5 [file jmir_v22i1e16249_app5.pdf]

	Alluvial Diagram	*Graphics*	*Concept Maps*	*System Map*
	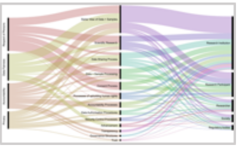	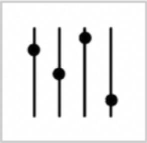	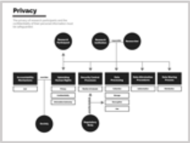	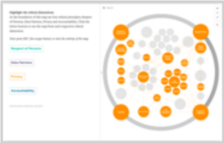
Function	Attention<br>Motivation<br>New Insight	Recall<br>Motivation	Attention<br>Motivation<br>New Insight<br>Elaboration	Attention<br>Motivation<br>New Insight Elaboration<br>Coordination
Attention: Is it visually engaging?	Yes	Yes	Yes	Yes
Context: Is the knowledge value clear?	No	Yes	Yes	No
Overview: Does it show the big picture?	No	No	Yes	Yes
Options to act: Can the knowledge be used?	No	No	No	Yes
Details: Is there enough detail appropriate?	No	No	Yes	Yes
Strengths	- Give an overview.<br>- Shows complexity.<br>- Relationships are clear.<br>- Visually appealing.	- Great for recall & attention.	- Ethical Principles are clear.<br>- Process is shown<br>- Relationships are clear.	- Playful and fun.<br>- Promotes exploration.<br>- Media enriches experience.
Weaknesses	- Limited information.<br>- Static image.	- Needs to be in context.	- Does not give the big picture.<br>- Static image.	- Limited to platform.<br>- Cannot customize.<br>- Functionalities not obvious to first time users.<br>- Context is not clear.
