# Supplementary material for: Visualizing an Ethics Framework: A Method to Create Interactive Knowledge Visualizations From Health Policy Documents
Source: J Med Internet Res. 2020 Jan 14;22(1):e16249. doi: 10.2196/16249 (PMC6996733; doi:10.2196/16249)

RESEARCHER | **PARTICIPANT** | **SOCIETY** | **INSTITUTION**

Part 1 | Part 2 | Part 3 | Part 4 | Part 5 | Part 6 | Part 7 | Part 8 | Part 9 | Part 10 | Part 11

1. WHAT DATA AND SAMPLES

We refer to all data types and samples that can be used in the context of health research.

This includes:

- Health-related personal data and samples that were not originally collected for research purposes.
- Data that are not conventionally associated with the practice of medical research: such as geolocation data, social media content, data from commercial portable sensors, and the like.

For definitions and examples of data these types, click the nodes on the map.

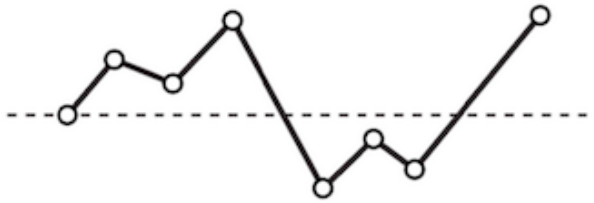

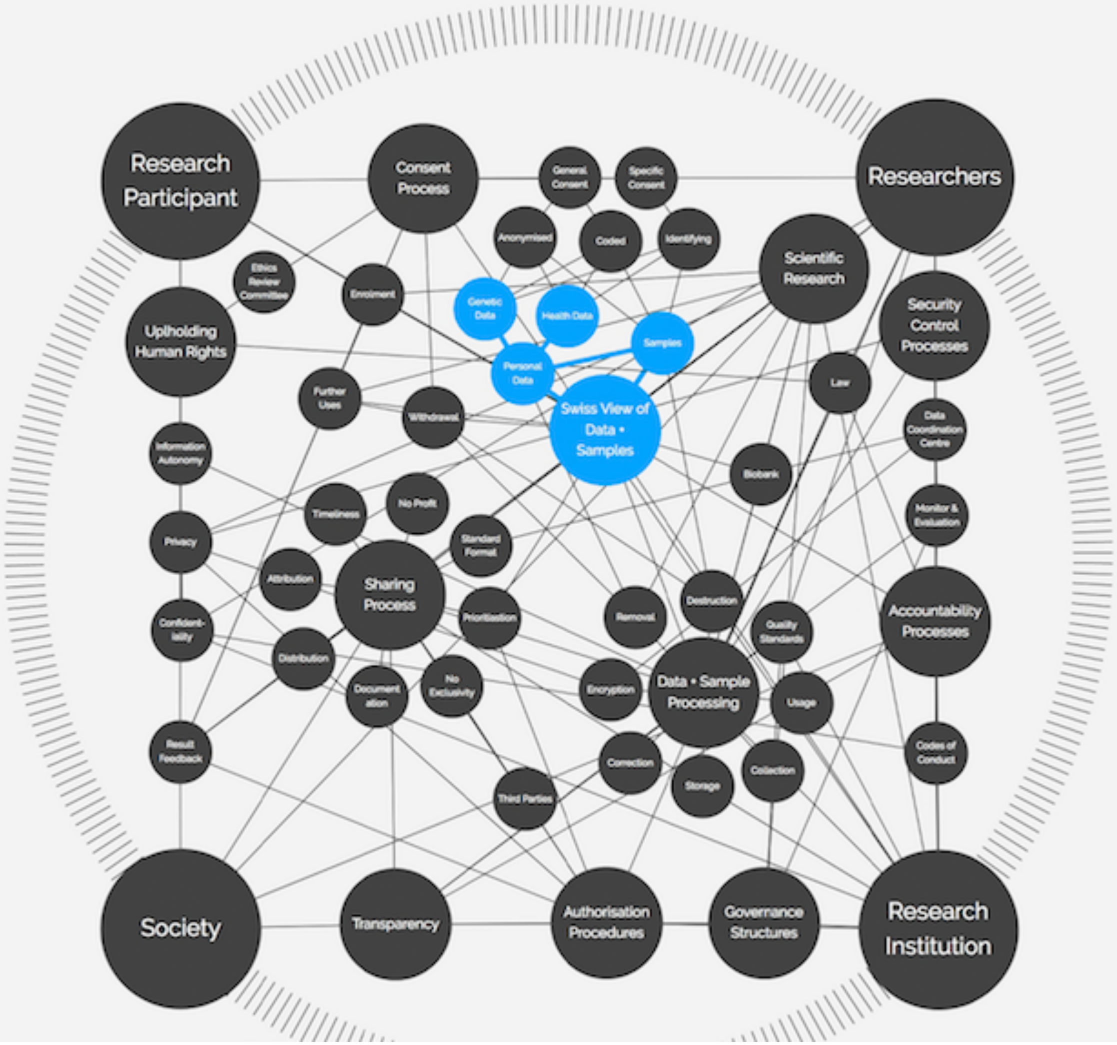

https://ethicalsystemsmap.hest.ethz.ch

HEALTH DATA ETHICS MAP

Navigating Health Data Ethics

About The Map

Researcher | Participant | Society | Institution

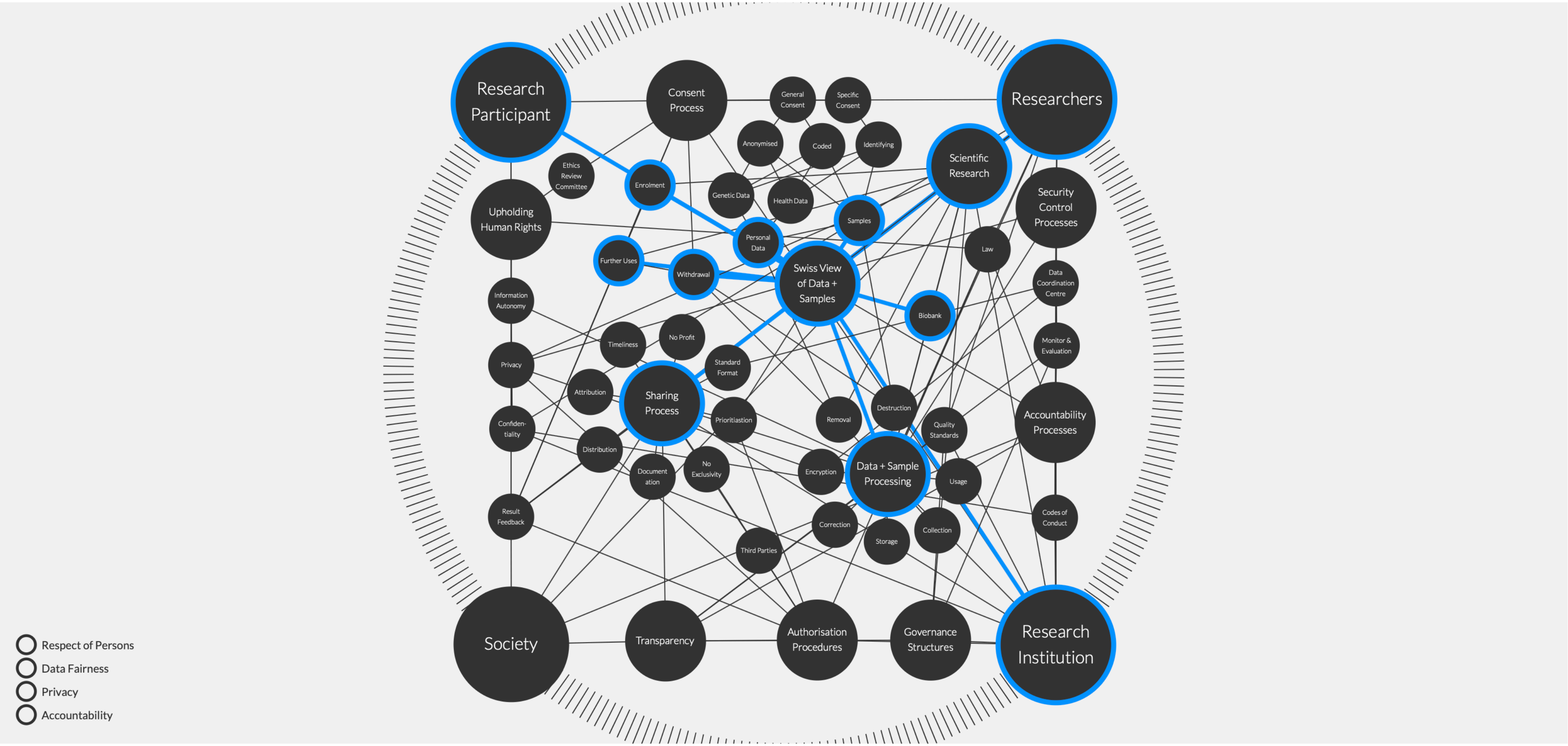

Navigating Health Data Ethics

5. Your Rights

You have human rights, enjoy intrinsic moral worth and have an entitlement to act as an autonomous person.

Your rights take precedence over the interests of scientific knowledge production. Your rights should be respected and protected by anyone processing your data or handling your samples, for any purpose and at any time.

To read more about how ethics review committees safeguard your rights, click the node on the screen.

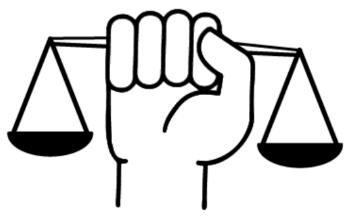

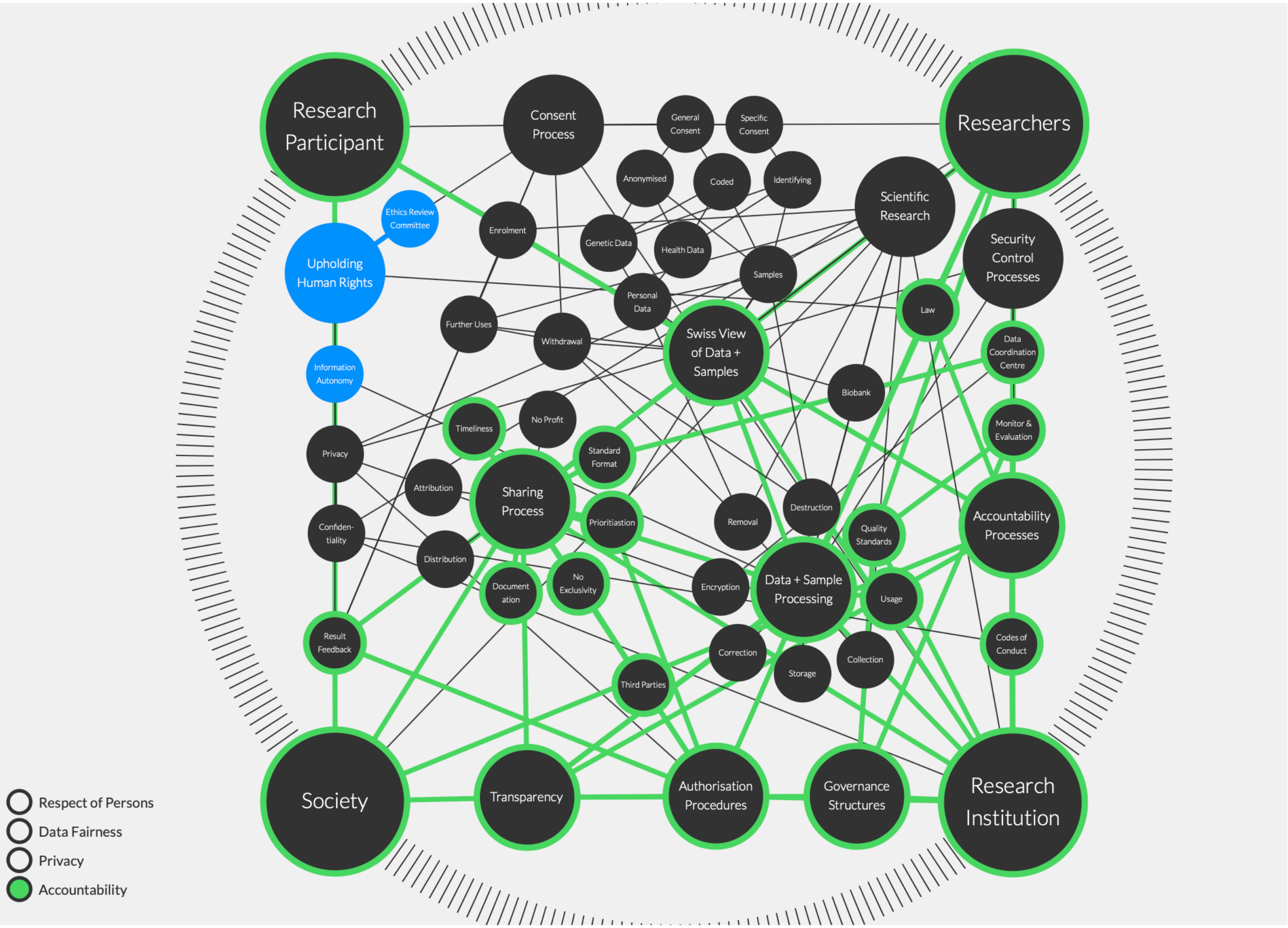

https://ethicalsystemsmap.hest.ethz.ch

HEALTH DATA ETHICS MAP

Navigating Health Data Ethics

About | The Map

Researcher | **Participant** | Society | Institution

Part 1 | Part 2 | Part 3 | Part 4 | **Part 5** | Part 6 | Part 7 | Part 8 | Part 9 | Part 10 | Part 11

5. Your Rights

You have human rights, enjoy intrinsic moral worth and have an entitlement to act as an autonomous person.

Your rights take precedence over the interests of scientific knowledge production. Your rights should be respected and protected by anyone processing your data or handling your samples, for any purpose and at any time.

To read more about how ethics review committees safeguard your rights, click the node on the screen.

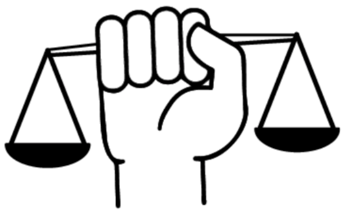

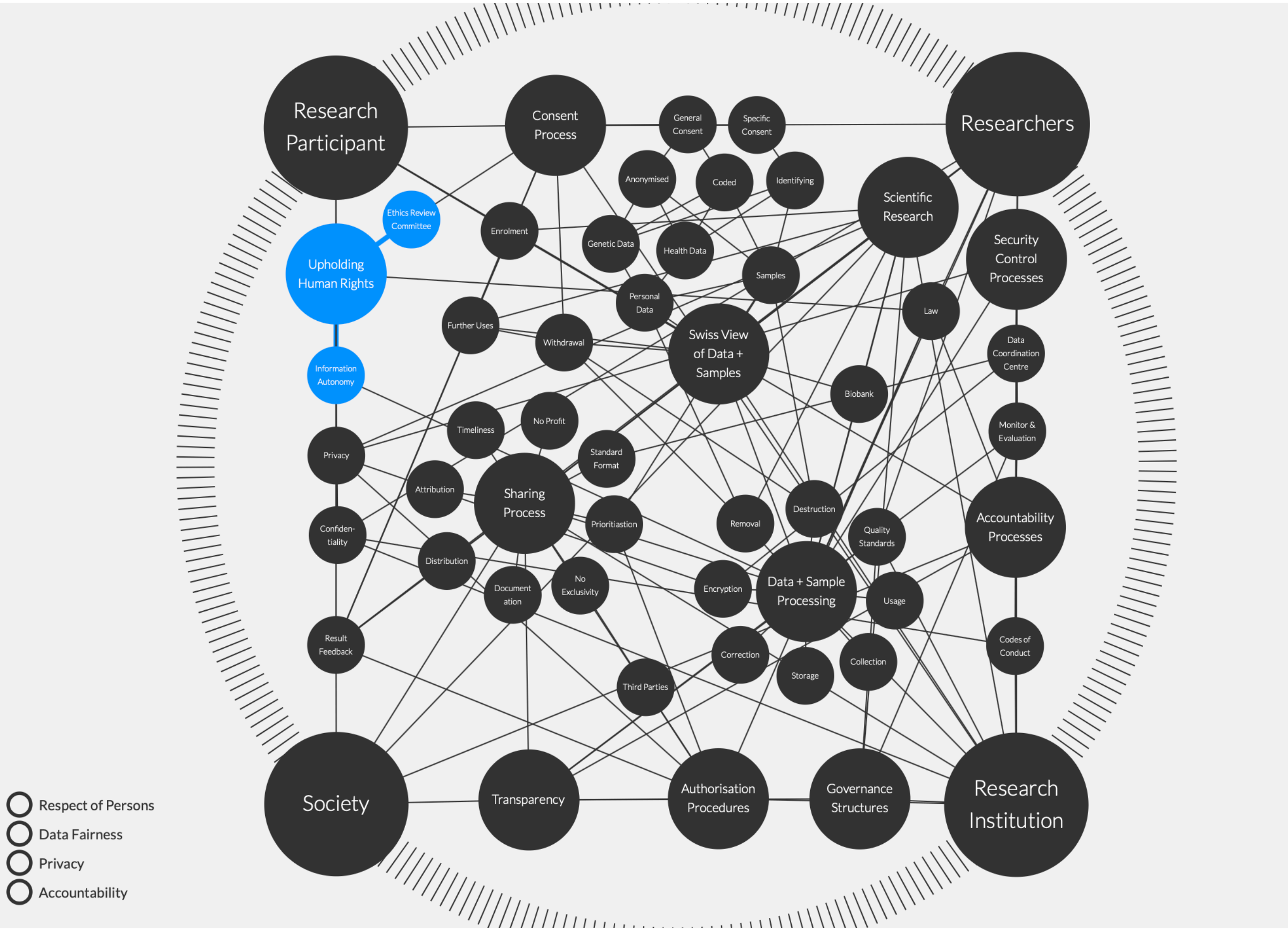

Supplement: Multimedia Appendix 6 [file jmir_v22i1e16249_app6.pdf]
